# Sepsis in Burns—Lessons Learnt from Developments in the Management of Septic Shock

**DOI:** 10.3390/medicina58010026

**Published:** 2021-12-24

**Authors:** Dorothee Boehm, Henrik Menke

**Affiliations:** Department of Plastic, Aesthetic and Hand Surgery, Specialized Burn Center, Sana Klinikum Offenbach, Starkenburgring 66, 63069 Offenbach, Germany; henrik.menke@sana.de

**Keywords:** septic shock, sepsis, burns, SOFA, antibiotic timing

## Abstract

After surviving the acute phase of resuscitation, septic shock is the cause of death in the majority of burn patients. Therefore, the management of septic shock is a cornerstone in modern burn care. Whereas sepsis therapy in general has undergone remarkable developments in the past decade, the management of septic shock in burn patients still has a long way to go. Instead, the differences of burn patients with septic shock versus general patients have been emphasized and thus, burn patients were excluded in every sepsis study which are the basis for modern sepsis therapy. However, due to the lack of evidence in burn patients, the standards of procedure for general sepsis therapy have been adopted in burn care. This review identifies the differences of burn patients with sepsis versus other septic patients and summarizes the scientific basis for modern sepsis therapy in general ICU patients and burn patients. Consequently, the results in general sepsis research should be transferred to burn care, which means the implementation of effective screening, early resuscitation, and efficient antimicrobial treatment. Therefore, on the basis of past developments and in the light of the current update of the Surviving Sepsis Campaign guidelines, this review introduces the “Burn SOFA score” and the “3 H’s of burn sepsis” as a screening tool for early sepsis recognition in burn patients.

## 1. Introduction

In the history of burn care there have always been two challenges that accounted for the majority of mortality in burn patients. Resuscitation and the acute phase of the first three days after burn trauma as the first critical situation have been optimized in the past and further developments are still increasing the survival rate even for severely burned patients [1]. Sepsis however, being the second significant challenge in burn care, is still the major cause of death after the first 24 h after trauma. Previous studies showed sepsis to be the cause of death in 50–60% of non-survivors [2,3,4]. In the literature, a wide range of sepsis prevalence, between 26% and 65%, is reported among burn patients depending on age and burn severity of the analyzed burn population and depending on the definition of “sepsis” [5,6,7]. Whereas the overall mortality is continuously decreasing for burn patients [8], the mortality of septic burn patients is still as high as 34% [7]. This underlines the importance of the topic for the patients’ outcome in burn care.

Though huge efforts were made in the past and multiple studies have enlarged the knowledge in the field of sepsis and septic shock in general, these studies have always excluded burn patients and thus the scientific status quo has changed only marginally over years. Despite—or because of—this lacking evidence clinicians have developed a very sensitive “gut feeling” for dynamic changes in their burn patients prior to meeting the classical criteria of sepsis. The clinical diagnosis by an experienced burn team is still a gold standard to define sepsis in burn patients [9,10].

This paper describes the differences of burn patients versus general septic patients and the scientific basis for actual clinical sepsis therapy. The current update of sepsis guidelines in 2021, as well as screening tools for the early diagnosis of sepsis, are discussed. Furthermore, the results of sepsis research will be transferred to burn care including the scientific data to develop a modified Sequential Organ Failure Assessment (SOFA) score as well as a screening tool for burn patients suspected of sepsis and septic shock.

## 2. Definitions of Sepsis/Septic Shock and Mortality in General Patients

In 1991 the first consensus conference published the Sepsis-1 definition along with the introduction of the systemic inflammatory response syndrome (SIRS) as a first approach to standardize the group of septic patients [11]. They defined sepsis as inflammatory response to an infection and subclassified sepsis into severe sepsis and septic shock. Though the criteria for SIRS were too nonspecific and complex, these definitions led to more standardized research and thus laid the foundation for following updates of the sepsis definition and modern treatment. The 2001 Sepsis Definitions Conference added multiple parameters and biomarkers to the definition of sepsis to describe the different stages more closely [12] but the definitions of SIRS, sepsis, and its different stages remained the golden standard until the Sepsis-3 definitions were published in 2016 [13]. The new definitions focused not on inflammation but on the organ dysfunction resulting from the host response to infection. Since this dysfunction is not a continuous process but rather a complex situation depending on co-morbidities and the patient’s history, a dynamic assessment of sepsis and the development of organ dysfunction was necessary. Thus, the term “severe sepsis” was abandoned and the organ dysfunction assessed by the SOFA score was emphasized with the new sepsis definition:

*“Sepsis should be defined as life-threatening organ dysfunction caused by a dysregulated host response to infection.”* [13]. Moreover, the definition of septic shock was updated. Meta-analysis and systemic review data showed a significantly higher mortality when both parameters—hypotension and lactate levels—were used to define septic shock [14]. Thus, septic shock should more closely define those septic patients with higher mortality. With this rationale, septic shock is now defined as sepsis with vasopressor therapy to maintain a mean blood pressure of at least 65 mmHg and increased lactate levels of ≥2 mmol/L [13,14].

Naturally, new definitions not only influence clinical treatment but also research. Peake et al. analyzed the scientific impact of Sepsis-3 definitions and found that most patients with positive Sepsis-2 criteria also fulfilled the Sepsis-3 definition [15]. Therefore, the group of sepsis patients in general has not significantly changed concerning in-hospital mortality after Sepsis-3 definition update, ranging between 18 and 20%. In contrast, a significantly lower number of patients met the criteria of septic shock in comparison to the former definition. Nevertheless, this smaller septic shock group showed a significantly higher mortality (approximately 30%) than the group of patients meeting the former criteria of septic shock (17%). The analysis of German ICUs revealed a prevalence of sepsis among general ICU patients of 12.6% [16,17]. The ICU mortality for patients meeting the Sepsis-2 criteria for severe sepsis and septic shock in German ICUs was 34% whereas patients meeting the Sepsis-3 definition of septic shock showed an increased mortality of 44%. In conclusion, future research needs to find the new “baseline mortality” for septic shock patients after the definition update. The development of sepsis definitions is summarized in Table 1.

## 3. Characteristics of Septic Burn Patients

The initial phase after burn injury is characterized by an enormous release of pro-inflammatory cytokines leading to systemic inflammatory response syndrome (SIRS). Typical cytokines such as interleukin 1 (IL-1), IL-6 and tumor necrosis factor α (TNF-α) regularly peak within the first 24 h after burn injury [18]. Furthermore, the metabolic rate of patients with major burns increases to 1.5–2 times compared to normal and can persist for several weeks followed by altered metabolism for months and years [19]. Within this hyper-metabolic state multiple vital signs, including temperature, respiratory rate, and heart rate, increase in burn patients which can be understood as a persistent SIRS. This renders the normal definition of SIRS not applicable to burn patients.

The mediators and cytokines in the acute phase also trigger the release of anti-inflammatory cytokines increasing continuously after the acute phase. In this phase, pro-inflammatory cytokines decrease and thus, anti-inflammatory cytokines dominate the clinical picture as “compensatory anti-inflammatory response syndrome” (CARS). The typical markers for CARS-among others-are IL 10 and transforming β (TGF-β) which peak approximately 1 week after burn injury. At the same time, the activity of natural killer (NK) cells which play a pivotal role in inflammatory response, and which are the prominent source of interferon γ (IFN γ), decreases significantly [20]. However, immunomodulation via IFN γ administration and IL-10 blocking has not yet shown any benefit [21]. Thus, the CAR-syndrome and the decline of NK cells as well as the lack of IFN γ may be the main but not the only causes for the immunosuppression that characterizes the severely burned patient.

Clinically, the immunosuppression of burn patients leads to an increased rate of infections and sepsis as well as a rapid onset from an extremely short “pre-septic” phase to septic shock. Therefore, severe burns are at risk for septic shock even before they meet the Sepsis-3 criteria. Herein, the main risk factors are age ≥ 50 years, inhalation injury and increasing TBSA, especially full-thickness burns ≥ 30% [5]. Taking the characteristics of septic burn patients into account, the lower mortality rate of sepsis in trauma patients (7–23%) and in general ICU patients (21–53%; burn patients: 28–65%) [22] and a 28-mortality rate of general medical and surgical ICU population of 17–33% [23,24,25] are unsurprising.

## 4. Sepsis Definitions in Burn Patients

Due to the different clinical signs and symptoms of beginning sepsis in burn patients, the Sepsis-2 definition was not applicable to burn patients. Therefore, the American Burn Association (ABA) published an expert consensus in 2007 [26]. This was the first sepsis definition specific to burn patients. Due to a more dynamic and more complex situation in burn patients, this definition uses more parameters to verify sepsis adding thrombopenia, enteral feeding intolerance and hyperglycemia to the classic parameters. As early as 1993 Housinger et al. showed that burn patients reliably present decreased thrombocyte counts before developing sepsis [27]. This phenomenon must be separated from the dilution phenomenon in the acute phase of burn trauma and thrombopenia and is therefore invalid as sepsis parameter before day 3 after burn injury. Enteral feeding intolerance was reported as a regular sign of sepsis in burned children [28] often associated with a distended abdomen. Hyperglycemia or insulin intolerance have also been shown to precede septic episodes in burn patients [29].

In a population of over 1000 burn patients, the SEPSIS-3-criteria showed superior sensitivity of 88.8% versus the ABA-criteria (84.6%) in defining sepsis but lower specificity with only 37.0% compared to the ABA-criteria (61.8%) [30]. Thus, the ABA definition showed acceptable performance in this retrospective study. However, ABA sepsis criteria have been criticized in the past and further studies tried to find a more reliable sepsis definition for burn patients [31], e.g., the BURN-6 criteria published in 2013 [32] or FF4 published 2018 [33]. In comparison to the ABA criteria, BURN-6 included hypotension (mean arterial pressure, MAP < 60 mmHg), presence of vasoactive medications and base deficit < −6 mEq/L. Other criteria, i.e., tachycardia, hypothermia and hyperglycemia were only redefined. A multivariable analysis of different parameters in a burn population with positive blood cultures showed the best performance of the combination of temperature (>39 or <36 °C), hypotension (MAP decrease ≥ 10%), tachycardia (>130 bpm) and gastric residual volume twice the actual feeding rate [33]. This FF4-model outperformed the BURN-6 and ABA-criteria in the analyzed burn population. In a retrospective study, the comparison of BURN-6, ABA-criteria and the Sepsis-3 definition clearly showed the superiority of the Sepsis-3 [34] to predict sepsis also in burn patients. Thus, the Sepsis-3 definition and therefore the SOFA score should be adopted for the population of burn patients in future research.

Furthermore, the ABA definition and positive blood cultures showed only weak correlation [31]. However, blood cultures remain negative in approximately 40% of clinically septic patients in a general ICU population [35]. Not to mention the necessary time to gain positive results, rendering an early diagnosis of sepsis impossible. Thus, blood cultures may confirm the diagnosis and guide de-escalation of antibiotic therapy, but they are of no avail in the initial decision-making.

As yet, no definition has been found that showed a higher sensitivity than the clinical diagnosis by an experienced burn team. This illustrates the difficulty to define sepsis in burn patients with adequate sensitivity to initiate sepsis therapy as early as possible but at the same time with an acceptable specificity to prevent over-treatment of non-septic patients.

## 5. Sepsis Marker and the (Burn-) SOFA Score

In search of a reliable sepsis marker, many biomarkers have been screened and analyzed in the past [36,37]. Herein, procalcitonin (PCT) and interleukin 6 (IL-6) are the most promising candidates. PCT as a pro-peptide of calcitonin increases 2–4 h after the release of endotoxins and pro-inflammatory mediators and peaks 24 h thereafter. Though some controversies exist in the literature, an increasing PCT of ≥1.5 ng/mL also shows an adequate sensitivity (82%) and specificity (91%) in burn patients [38]. However, severely burned patients show high PCT values post burn with a continuous decline in the following days [39]. Similarly to the majority of sepsis markers, PCT has only limited use within the acute phase after major burn injury. The initial phase after trauma excluded, a recent meta-analysis showed [40] a combined sensitivity of 67% and combined specificity of 87% in predicting septic episodes in burn patients. Comparably, IL-6 can be used as sepsis marker to differentiate between inflammation and blood stream infection. In a recent retrospective study, IL-6 showed a high correlation with positive blood cultures [41]. Whereas IL-6 was elevated in all burn patients, IL-6 levels were significantly higher in those patients with positive blood cultures. Thus, blood stream infections could be predicted with adequate reliability (SROC 0.7; sensitivity 79.5%, specificity 56.5%). This emphasizes the importance of positive blood cultures in the treatment of septic patients though the SEPSIS-3 definition reasonably depreciated blood cultures to define sepsis.

Beside biomarkers for sepsis diagnosis, the SOFA score has gained increasing popularity and is now the leading diagnostic tool for sepsis definition and treatment. Since most biomarkers need some time to gain positive values, dynamic changes in organ function and even organ failure can precede positive sepsis markers, especially in rapid development of septic shock. The phenomenon of sequential organ failure was described as early as the 1970s [42] and especially linked to uncontrolled infections [43]. Different scores were published to define and grade failure of different organ systems, since organ failure is not an on/off-phenomenon but rather a dynamic continuum from mild dysfunction to severe failure. As a compromise between a detailed score for various organs and a score that is applicable on every ICU, the SOFA score was developed on the basis of a consensus meeting in 1994 [44]. The score was evaluated in a prospective multi-center study in critically ill patients (n = 1449) in 16 countries [45]. To define and detect the development of sepsis according to the SEPSIS-3 definition, an increase in the current SOFA score of ≥2 points is used as cut-off value. Therefore, the SOFA score should be assessed daily for an early detection of sepsis.

In burn patients, the SOFA score has so far been analyzed in a few studies [7,46,47]. A retrospective study on 169 burn patients showed an excellent correlation of the SOFA score and mortality [47]. Herein, the respiratory, cardiovascular and haematologic components of the score had the highest correlation with 30-day outcome followed by the renal system. The Glasgow Coma Scale (GCS) and the hepatic parameter showed no contribution to the performance of the score in the first 24 h after admission. Unfortunately, the authors did not comment on sedation regimes and the assessment of the GCS in the studied burn population. As a tool for early sepsis diagnosis in burn patients, Belba et al. showed a good correlation between the SOFA score and mortality in burn patients with sepsis [7]. In this study, SOFA scores were assessed at 3, 7, 14 and 21 days after burn injury. The change in SOFA scores of ≥2 points between the different time points, especially the difference between day three and day seven, enabled a reliable discrimination between survivors and non-survivors. Therefore, the trend of sequential SOFA scores and changes of ≥2 points in the daily assessment of the SOFA score could indicate an adequate treatment or show newly developing sepsis as a reason for the deterioration of organ dysfunction also in burn patients.

Taking these results and the ABA-criteria for sepsis into account, it seems to be logical to use a modified SOFA score for burn patients. A modification of the former Multiple Organ Dysfunction (MOD) score has been published as early as 1993 [48] but has never gained popularity due to practical issues, whereas the SOFA score with its simplified parameters has been adopted into clinical use in general ICUs worldwide. Therefore, it is an intriguing point to modify the SOFA score to the specific characteristics of burn patients. This Burn SOFA score should meet the following expectations:

(1) Grade organ dysfunction as a continuum;
(2) Use parameters that can be assessed easily in every ICU (worldwide);
(3) Prefer fast reacting parameters/organ systems due to the fast onset of septic shock in burn patients;
(4) Reliably assess organ dysfunction in sedated as well as in non-sedated burn patients;
(5) Show high sensitivity as a screening tool and show adequate specificity to indicate adequate treatment or further deterioration of the patient.

Whereas the original SOFA score meets the first and second point perfectly, the slow reacting hepatic component, i.e., bilirubin levels, are not applicable to burn patients and their rapid onset to septic shock. Furthermore, bilirubin levels are not frequently measured in burn patients [47]. As a substitute, the hyperglycaemia frequently preceding septic changes in burn patients could be used as proposed by the ABA in their sepsis definition. The fourth point questions the GCS as the neurologic component of the SOFA score since GCS assessment is often inaccurate in sedated patients. We therefore propose the use of GCS for non-sedated burn patients. The difficulty in the correct grading of the GCS in sedated patients is not specific to burn patients but a difficulty in all intensive care patients. However, the rapid onset to septic shock is a characteristic of burn patients leading to deterioration not within days but in case of wound sepsis sometimes within a few hours. Thus, the timely diagnosis is of utmost importance in this population, and it is therefore reasonable to exchange an unreliable parameter, i.e., the GCS in sedated patients, for a parameter that is easy to assess in sedated patients. However, in non-sedated patients we still recommend the GCS which is a fast-reacting parameter also in burn patients. In contrast, mechanical ventilation and sedation also asks for gastric tubing and early and aggressive enteral feeding in burn patients due to their increased metabolic rate. Thus, we propose feeding intolerance as alternative parameter for ventilated and sedated patients. Though increasing feeding intolerance is seen frequently in burn patients developing septic episodes, it is a very unspecific parameter of the gastro-intestinal organ system. Unfortunately, another complication of burn resuscitation causes similar clinical signs. The abdominal compartment syndrome develops in severely burned patients at different time points [49] and is correlated with mechanical ventilation with high ventilation pressures especially [50]. Therefore, an onset of intestinal dysfunction also asks for further monitoring and at least the assessment of bladder pressures.

Concerning the cardiovascular system, vasopressor therapy has changed since the first introduction of the original SOFA score. In burn patients, persisting tachycardia often rules out the use of dobutamine. Thus, nor-/epinephrine is the first choice to treat hypotension in burn shock as well as septic shock. We therefore propose a modification of the grading of cardiovascular dysfunction (Table 2).

## 6. SSC Guidelines and the Treatment of Sepsis and Septic Shock

The prognosis of septic patients in the general ICU has been devastating over the years. Increasing the prognosis of septic patients was the designated aim of the Surviving Sepsis Campaign (SSC). Within this challenge and still ongoing struggle the landmark study of Rivers et al. [51] was a ground-braking publication that focused on the correlation of the patient’s prognosis and the beginning of antibiotic therapy as well as a consequent, early-goal-directed therapy. The effect of an early goal-directed therapy on patients’ outcome has been questioned in three large RCT studies [52,53,54] and one meta-analysis [55]. Whereas these studies have seen no difference in patients with early goal directed therapy in comparison to patients with standard care, they confirm the success of the publication of Rivers and the work of the SSC. Though Rivers first published an early goal-directed therapy and propagated the achievement of these goals within the first 6 h after sepsis diagnosis, the main issue of his study were the screening and diagnosis of septic patients in the emergency department (ED) and the early aggressive resuscitation of these patients including early antimicrobial therapy within the first three hours. Since these cornerstones of sepsis treatment-sepsis screening, early resuscitation and short time-to-antibiotics-became standard of care in sepsis treatment, the achievement of specific goals, especially central venous or mixed-venous saturation became secondary [56]. Thus, the first guidelines of the SSC in 2004 adopted the results of Rivers but were refined in the following updates [57,58,59]. Since 2004, the focus shifted from specific goals to guide sepsis treatment to an effective screening for septic patients and training of medical personnel to enable resuscitation as early as possible and the various recommendations of the first guidelines were merged into sepsis bundles to be achieved in 3 h and 6 h in the second update in 2012 [59]. With an increasing awareness of septic patients and the compliance to the suggested sepsis bundles, sepsis mortality significantly decreased [60] and emphasized the importance of an effective sepsis screening tool. Hence, the third update of the SSC guidelines proposed the quick SOFA score as defined by the SEPSIS-3 definitions as screening tool for patients in the Emergency Department/in pre-ICU settings [13,61]. Recent studies have analyzed the performance of this quick SOFA as a screening tool and found a positive quick SOFA only in 24% of septic patients [62]. Though, the patients with positive quick SOFA also showed the highest mortality rate, the quick SOFA revealed an insufficient sensitivity as a screening tool. Consequently, the actual SSC guidelines published recently recommended against the quick SOFA as a single screening tool and suggested additional scores or the use of lactate to enhance the sensitivity of the quick SOFA score [63].

Initially, a normalization of lactate levels was recommended by the SSC to be achieved as early as possible [59]. Various studies proofed the reliability of lactate levels to evaluate the patient’s mortality rate and response to treatment and to guide sepsis resuscitation [64,65]. Recent studies also showed adequate performance of lactate as a screening tool for patients with suspected sepsis [66,67,68]. The combination of a screening score with lactate levels could therefore meet the criteria for an adequate sepsis screening tool and should be analyzed by future studies.

The second cornerstone of sepsis therapy is early resuscitation using crystalloids as first line with a rate of 30 mL/kg within the first 3 h [61,63]. Due to lacking evidence the guidelines suggest the additional use of colloids without a clear recommendation [59]. To avoid fluid overload the SSC now recommends against the original early goal directed therapy including static parameters as the central venous pressure. Instead, dynamic parameters are strongly recommended in the actual SSC guidelines, since they are superior in dynamic situations such as sepsis and even more so in septic shock. Dynamic parameters include the assessment of stroke volume and cardiac output also combined with fluid challenge or passive leg raise maneuver. In this context, point-of-care ultrasound, and especially echocardiography, has gained importance over the last years [69]. The mean arterial pressure (MAP) as the last remaining parameter of the original early goal-directed therapy, has been corrected, i.e., the resuscitation and vasopressor therapy should target a MAP of 65 mmHg. The lower target showed no adverse affects even in elderly septic patients but enabled to cut down vasopressor requirements [70,71].

The antimicrobial therapy as the third and most crucial keystone of sepsis treatment was also refined in the recent guideline update. In septic shock patients, antimicrobials should be initiated immediately and without a delay by diagnostic measures such as obtaining blood cultures. Whereas past guidelines recommended drawing blood cultures before starting antibiotics, blood cultures became secondary since a significant part of septic patients show negative blood cultures [35]. Thus, the time-to-antibiotics should be as short as possible because early antibacterial treatment increases survival rates [72,73]. An observational study even showed an increase of 1.5% in mortality for every hour delay in intensive treatment including antibiotic therapy [74]. A more complex issue is the right dosing of antimicrobial therapy, i.e., adjusting the individual therapy to the patient’s situation. Since sepsis changes hepatic metabolism and renal clearance of antibiotics, whereas resuscitation leads to increased distribution volume and hypoalbuminemia alter the pharmacokinetic of several antibiotics, an individual adjustment of the antimicrobial therapy is necessary. Herein, the use of therapeutic drug monitoring is pivotal but not available in many settings. Though a one size fits all approach can not be recommended, the dosing should be checked closely, especially during the use of renal replacement therapy or extracorporeal membrane oxygenation or other replacement therapies. However, concerning beta-lactam antibiotics the delivery of a loading dose as bolus before the start of prolonged infusions is recommended. Thus, therapeutic levels can be achieved without delay [75,76].

## 7. Sepsis Guidelines and Sepsis Screening on Burn ICU

Due to the typical time course of sepsis in burn patients with fast deterioriation, screening for sepsis is highly important to enable early sepsis therapy. The characteristic causes of burn sepsis are either pneumonia and respiratory failure with an incidence of 20–56% [47] or septicemia due to wound infection (incidence approximately 50%) [77] with rapid onset of sepsis and haemodynamic dysfunction. Hence, a screening tool for burn sepsis should include the respiratory and cardiovascular organ system. The clinical symptoms of cardiovascular dysfunction at an early stage are provoked by relative hypovolaemia due to peripheral vasodilation and decreasing systemic vascular resistance. The clinical signs may be tachycardia and decreasing systolic pressure and is easily identified by an assessment of the volumetric status. The most sensitive parameter for hypoxia is the P/F-ratio which can also be estimated with oxygen gas flow and peripheral saturation. Clinical signs are tachypnoea or desaturation. Rather, the most crucial point is to evaluate the tendencies and changes over time than a certain cut-off value concerning these clinical signs and symptoms. Whereas organ failure should be assessed in a grading system, a sepsis screening tool should be ready to use in a wide range of burn patients and be positive or negative to enhance decision making for further diagnostic or immediate antimicrobial treatment. Therefore, we suggest a combination of three parameters, including the unspecific parameter of hypo- or hyperthermia as also proposed by the ABA consensus conference [26]. Since the course of septic onset at a very early stage especially is extremely variable, we propose rather a variety of clinical signs as a variable combination than a distinct parameter for each organ system (Table 3).

Thus, the focus rather lies on the main issues of beginning organ failure than on the variable clinical symptoms. These main issues are Hypoxia, Hypovolaemia, hypo-/Hyperthermia. The 3 H’s of burn sepsis could serve as a screening tool if at least 2 of the3 main issues appear without a clear non-infectious cause.

Furthermore, the time course of a suspected septic episode is crucial since high mortality is associated with a rapid deterioration. In general ICU patients, blood lactate has been proofed to be a reliable marker to select those patients at risk for a rapid onset of septic shock. In burn patients, elevated blood lactate is already an established indicator for increased morbidity and mortality [78] and Herero et al. proposed a cut-off value of ≥2 mmol/L [79]. A recent retrospective study showed increased lactate levels to be associated with increased rates of sepsis [80]. Consequently, blood lactate should be used both as additional parameter within the screening process and as prognostic factor to evaluate the patient’s response to the initiated therapy as claimed in the current SSC guidelines (SSC 2021).

## 8. Conclusions

To summarize the developments of sepsis definition and treatment in general ICU patients, the focus has shifted from clear definitions and clearly defined goals to be achieved to taking action and starting resuscitation and antimicrobial therapy as early as possible. The perfect sepsis definition or sepsis biomarker may never be found but the variable signs and symptoms of developing sepsis rather call for sensitive screening tools for patients at risk and dynamic parameters to guide sepsis treatment than for rigid definitions and targets.

This development conveniently fits in the standards of burn care, since burn patients characteristically show a myriad of signs and symptoms before they show a more rapid onset and organ deterioration compared to general ICU patients. Therefore, it is crucial to implement effective screening, early resuscitation, and efficient antimicrobial treatment into burn care likewise. To meet the specific concerns of burn patients, several modifications of existing screening tools and organ dysfunction scores may be necessary. In this review we therefore suggest the Burn SOFA score and the 3 H’s of burn sepsis to be used on burn ICU. The suggested Burn-SOFA as well as the 3 H’s of burn sepsis are based on the current literature and in accordance with the current ABA sepsis criteria and guidelines. Therefore, the effectiveness and performance of these tools must be analyzed in future studies.

## Figures and Tables

**Table 1:** Development of sepsis definitions and parameters to detect and define sepsis.

**Sepsis-1/1991 [11]**	Sepsis = SIRS as a response to infection (SIRS = systemic inflammatory response syndrome) defined by ≥2 parameter:
Parameter	>38 °C or <36 °C; heart rate > 90/min; respiratory rate > 20/min or PaCO_2_ < 32 mmHg; white blood cell count > 12,000/µL or <4000/µL or >10% immature forms
**Sepsis-2/2001 [12]**	no change in definition, but additional parameters to detect sepsis and organ dysfunction:
Parameter	significant edema/Positive fluid balance; hyperglycaemia in the absence of diabetes; C-reactive protein 2× above normal; procalcitonin 2× above normal; arterial hypotension; mixed venous saturation > 70%; arterial hypoxemia; acute oliguria, creating increase ≥ 0.5 mg/dL; coagulation abnormalities, thrombocytopenia; ileus; hyperbilirubinemia; hyperlactatemia, decreased capillary refill
**Sepsis-3/2016 [13]**	*“Sepsis should be defined as life-threatening organ dysfunction caused by a dysregulated host response to infection.”*
Parameter	increase in SOFA-score ≥ 2 points

**Table 2:** Burn SOFA score: Increases of ≥2 points could indicate deterioration of organ failure. **bold letters**: modification compared to the original SOFA score.

	Organ System/Parameter	1 Point	2 Points	3 Points	4 Points
	Respiratory/ PaO_2_/FiO_2_ [mmHg]	<400	<300	<200 with respiratory support	<100 with respiratory support
	Cardiovascular/ Hypotension	MAP <70 mmHg	**nor-/epinephrine ≤ 0.05 µg/kg/min**	**nor-/epinephrine ≤ 0.1 µg/kg/min**	**nor-/epinephrine ≥ 0.1 µg/kg/min** **or multiple vasopressors**
	Coagulation/ Platelets [×10^3^/mm^3^]	<150	<100	<50	<20
	Renal/ Creatinine or urine output	1.2–1.9 mg/dL (110–170 µmol/L)	2.0–3.4 mg/dL (171–299 µmol/L)	3.5–4.9 mg/dL (300–440 µmol/L) or <500 mL/day	≥5 mg/dL (>440 µmol/L) or <200 mL/day
	**Metabolism/** **Hyperglycaemia** (without history of diabetes mellitus)	**plasma glucose > 200 mg/dL (untreated)**	**>25%/24 h increase of insulin/h i.v.drip**	**>50%/24 h increase of insulin/h i.v.drip**	**persistent plasma glucose > 200 mg/dL** despite insulin bolus + continuous therapy
**patient non-sedated**	CNS/Glasgow Coma Scale (points)	13–14	10–12	6–9	<6
**patient sedated**	**intestines/enteral feeding intolerance**	**distended abdomen**	**gastric residual volume of 100% of feeding rate**	**gastric residual volume of 200% of feeding rate or inability of gastric feeding > 24 h**	**inability of gastric feeding > 48 h**

**Table 3:** The 3 H’s of burn sepsis.

Organ System	Main Issue	Clinical Signs	Additional Parameter
Respiratory	**Hypoxia** (impaired gas exchange)	tachypnoea, dyspnoea, desaturation, increasing O_2_-flow/FiO_2_	decreasing PO_2_/FiO_2_-ratio, radiologic signs of pneumonia?
Cardiovascular	**Hypovolaemia**	increasing tachycardia, decreasing systolic pressure, swinging arterial pressure curve	volumetric status: increasing respiratory variability (inferior vena cava), decreasing stroke volume/ventricular filling; decreasing systemic vascular resistance
Body Temperature	**Hypo-/** **Hyperthermia**	<36.5 °C >39.0 °C	routine microbiologic screening: causative agents?
≥2 criteria positive without other apparent cause = sepsis screening positive			
**-> measure blood lactate**			
**≤2 mmol/L**: increasing BurnSOFA score? -> consider sepsis and search for possible source and causative agents, -> check additional parameters and inflammatory biomarkers (PCT, IL-6) -> start sepsis treatment if additional parameters indicate sepsis			
**≥2 mmol/L** (increasing lactate level without other detectable cause): **initiate sepsis bundle** **without further delay!** -> start antimicrobial treatment within 45 min -> begin with antibiotic bolus to gain therapeutic levels -> discontinue antibiotics if non-septic causes show higher probability			

## Data Availability

Not applicable.
